# High-performance supercapacitor based on tungsten oxide iodide/polymer nanocomposite for advanced energy storage

**DOI:** 10.1038/s41598-025-29780-y

**Published:** 2025-12-04

**Authors:** Ahmed H. AbdEl-Salam, Hassan A. Ewais, Mohamed Rabia, Min Liu, Yasser M. Al Angari

**Affiliations:** 1 Chemistry Department, College of Science, University of Jeddah, Jeddah, Saudi Arabia; 2 Chemistry Department, Faculty of Science, King Abdulaziz University, P.O. Box. 80200, 21589 Jeddah, Saudi Arabia; 3 Nanomaterials Science Research Laboratory, Chemistry Department, Faculty of Science, Beni-Suef University, Beni-Suef, 62514 Egypt; 4 School of Physics, Central South University, Changsha, 410083 China

**Keywords:** Pseudo supercapacitor, Tungsten oxide iodide, Poly-1H pyrrole, Energy storage, Nanocomposite, Chemistry, Energy science and technology, Materials science, Nanoscience and technology

## Abstract

A novel pseudosupercapacitor has been successfully developed based on a nanocomposite of tungsten oxide iodide integrated with poly(1H-pyrrole) (WO_3-X_I_X_/P1HP). This hybrid material synergistically combines the redox activity of transition metal oxides with the conductivity and flexibility of a polymer matrix, enabling high-performance energy storage. The incorporation of iodide within the crystalline tungsten oxide framework enhanced electrochemical functionality, while scanning electron microscopy reveals cauliflower-like nanostructures (~ 30 nm) with hierarchical roughness, offering increased surface area for charge storage. As a result, the composite exhibited an outstanding capacitances of 775 F/g at 1.0 A/g and 425 F/g at 2.0 A/g, respectively. Corresponding energy densities reached 100 Wh/kg and 52 Wh/kg, respectively. These impressive values are attributed to the synergistic effect of efficient redox activity of the tungsten oxide iodide component, as well as the enhanced electron transport via P1HP matrix. The integrated hybrid architecture also ensures structural integrity and cycling stability. Overall, the WO_3-X_I_X_/P1HP nanocomposite represents a promising candidate for next-generation supercapacitors. Its superior electrochemical performance, structural robustness, and scalable synthesis suggest significant potential for practical applications in portable electronics and advanced industrial energy storage systems.

**Supplementary Information:**

The online version contains supplementary material available at 10.1038/s41598-025-29780-y.

## Introduction

Conducting polymers such as (P1HP) and polyaniline (PANI) have emerged as promising electrode materials for pseudosupercapacitors due to their intrinsic advantages that align well with the growing demand for cost-effective, high-performance energy storage systems. These polymers possess a unique combination of properties including high electrical conductivity, ease of synthesis, and relatively low environmental impact, making them particularly attractive for scalable supercapacitor applications. Their cost-effectiveness further amplifies their suitability for widespread industrial adoption, particularly in areas where sustainable and economically viable solutions are essential^1–3^.

Among various transition metal oxides explored for energy storage and sensing applications, tungsten oxide (WO₃) stands out due to its favorable electrochemical characteristics and chemical stability^4–7^. Similarly, P1HP, a widely studied conjugated polymer, is considered one of the most efficient pseudocapacitive materials. With a typical electrical conductivity in the range of 10–100 S/cm, P1HP provides a high charge transport rate, and its simple polymerization process makes it compatible with large-scale device fabrication. The operational voltage window of P1HP is also suitable for supercapacitor configurations, contributing to its overall efficiency and performance. These features make it highly applicable in energy storage devices, particularly in asymmetric supercapacitor configurations where both energy and power densities are critical^8,9^

Despite these advantages, one of the primary limitations associated with conducting polymer-based supercapacitors is their poor cycling stability. During repeated charge and discharge cycles, polymer electrodes undergo substantial volumetric changes as ions from the electrolyte are inserted and extracted. This ion doping and de-doping process leads to expansion and contraction of the polymer structure, respectively. Over time, these repetitive mechanical stresses result in structural degradation, fragmentation, and eventual loss of electrochemical activity. The inability of most polymer matrices to accommodate these volume changes compromises both their mechanical integrity and capacitive performance, significantly limiting their long-term application in practical devices^10^.

To address this limitation, the incorporation of metal oxide components into conducting polymer matrices has been explored as a strategic solution to improve structural and electrochemical stability^11,12^. Composites comprising conducting polymers and metal oxides offer synergistic benefits. Firstly, metal oxides such as tungsten oxide can act as physical buffering layers that mitigate the volumetric stress experienced by the polymer during cycling. This buffering effect can minimize cracking and degradation, thereby prolonging the electrode’s lifespan. Secondly, metal oxides can serve as conductive scaffolds that facilitate electron and ion transport throughout the composite structure. Even if some degree of polymer fragmentation occurs, the metal oxide network can maintain electrical connectivity and mechanical cohesion, thus preserving the functionality of the electrode^13,14^. Several studies have demonstrated the effectiveness of this composite approach in enhancing electrochemical performance. For instance, polymer-coated metal oxide electrodes have shown improved cyclic stability in lithium-ion batteries, largely due to the oxide’s ability to accommodate structural changes during ion insertion and extraction. The formation of hybrid materials—where the advantages of high-capacity metal oxides are combined with the flexibility and conductivity of organic polymers—offers a promising path toward the design of next-generation pseudosupercapacitors.

Recent research efforts have explored a variety of such hybrid structures for supercapacitor applications. Examples include MnO_2_–Mn_2_O₃/poly(2-methylaniline)^15^, polyaniline/silver oxide/silver^16^, CoO–CuO/g-C₃N_4_^17^, and Fe_2_O_3_/poly(2-aminothiophenol) composites^9^. While these systems represent innovative approaches to hybrid material design, their electrochemical performance often remains suboptimal. In many cases, specific capacitance (Cs) values are limited to around 70 F/g at low current densities (e.g., 0.2 A/g), which falls short of the levels required for competitive energy storage technologies. These constraints underline the urgent need for further material development aimed at significantly enhancing specific capacitance, energy density, and power output.

In this study, a novel WO_3-X_I_X_/P1HP nanocomposite was synthesized and subsequently utilized to fabricate an advanced pseudosupercapacitor system, which was thoroughly evaluated using a three-electrode setup. The electrochemical performance of the device was assessed through galvanostatic charge–discharge (GCD) measurements across current densities ranging from 1.0 to 5.0 A/g, as well as cyclic voltammetry (CV) conducted at scan rates between 50 and 300 mV/s. Key electrochemical parameters, including energy density (E), power density (P), and specific capacitance (Cs), were systematically calculated to determine the capacitor’s efficiency and functionality. The exceptional electrochemical behavior demonstrated by this hybrid supercapacitor underscores its strong potential for industrial applications. Ongoing work by our research team focuses on advancing this system toward a scalable prototype for future practical deployment in energy storage technologies.

## Experimental part

### Materials and devices

1H-pyrrole (99.9%) was sourced from Across, USA, while sodium tungstate (Na_2_WO_4_, 99.9%) was supplied by VWR, Germany. Potassium persulfate (K_2_S_2_O₈, 99.8%), graphite powder (99.9%), and acetic acid (CH_3_COOH, 99.9%) were procured from Pio-chem, Egypt. Additionally, nafion (5%) and ethanol (99.95%) were purchased from Sigma Aldrich, USA. Hydrochloric acid (HCl, 36%), iodine (I_2_, 99.9%), and potassium iodide (KI, 99.95%) were obtained from El Nasr Co., Egypt.

The morphological and structural characterizations were performed using SEM (Zeiss), TEM (Jeol), XPS (Kratos), XRD (X'Pert), and FTIR (Bruker). These advanced analytical instruments provided comprehensive insights into the surface texture, crystalline structure, elemental composition, and chemical bonding of the synthesized WO_3-X_I_X_/P1HP nanocomposite.

### Synthesis of WO_3-X_I_X_/P1HP nanocomposite

The synthesis of the WO_3-X_I_X_/P1HP nanocomposite is carried out in two sequential steps, beginning with the oxidation of 1H-pyrrole and followed by its reaction with sodium tungstate (Na_2_WO_4_). The first step involves dissolving the monomer (1H-pyrrole) in acetic acid, where the monomer and acetic acid concentrations are maintained at 0.05 M and 0.14 M, respectively. The oxidation process is facilitated using iodine/potassium iodide (I_2_/KI) as the oxidizing agent. During this stage, the polymeric network of P1HP undergoes substantial iodide insertion, leading to the formation of the intermediate I_2_-P1HP. This step is crucial as it enhances the structural and electronic properties of the polymer, making it more receptive to further modifications.

**Fig. 1:**
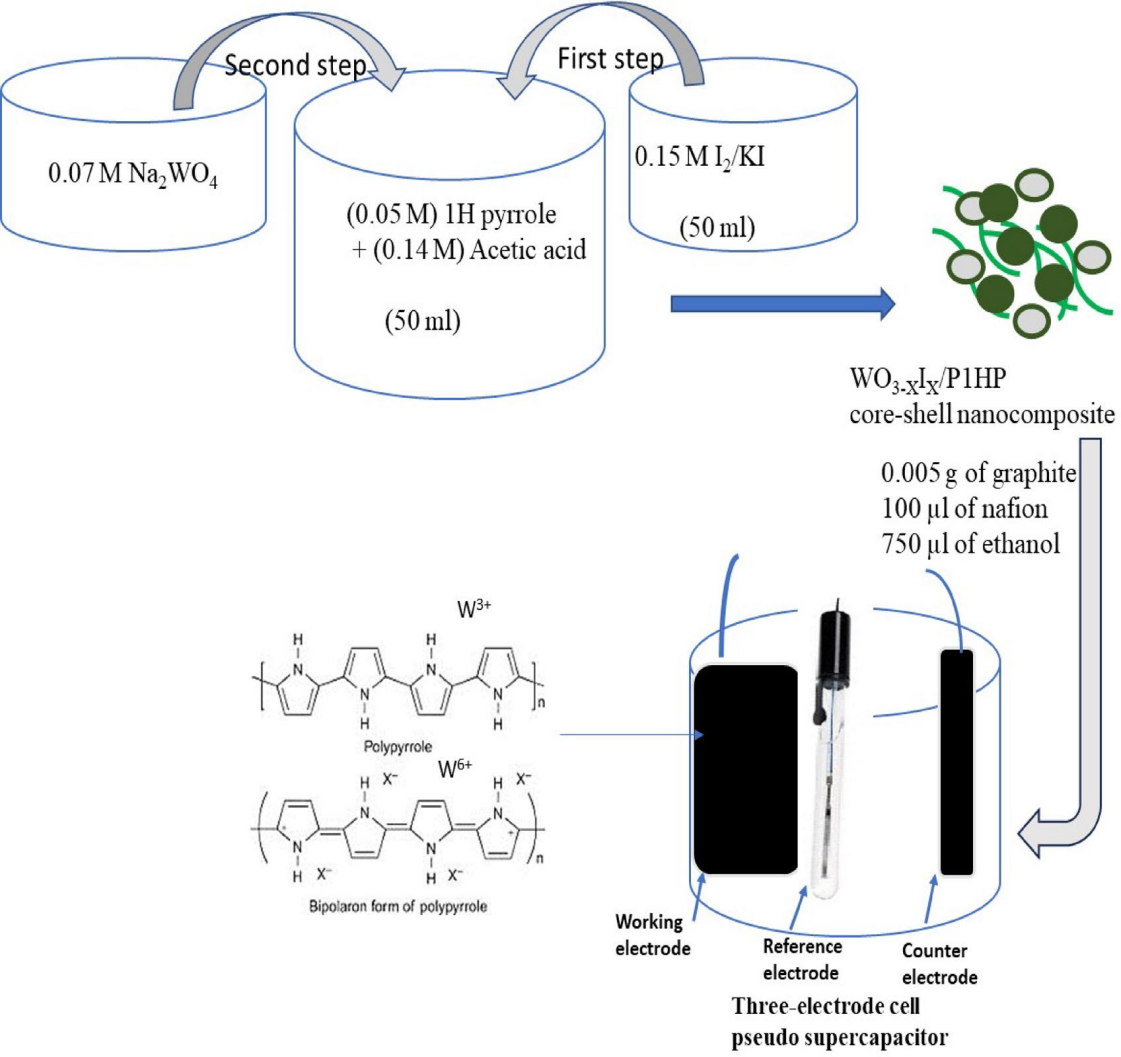
The fabrication of the pseudo supercapacitor based on WO_3-X_I_X_/P1HP nanocomposite.

In the second stage, the preformed I_2_-P1HP is subjected to a reaction with Na_2_WO_4_. This interaction follows a double displacement reaction mechanism, facilitating the successful incorporation of WO_3-X_I_X_ within the polymer matrix. The resulting nanocomposite, WO_3-X_I_X_/P1HP , is expected to exhibit promising electrical properties due to the effective integration of the tungsten oxide phase into the polymer framework. The introduction of WO_3-X_I_X_ within the P1HP matrix significantly enhances the material’s conductivity, charge transfer efficiency, and overall electrochemical performance (Fig. 1).

For comparison and further assessment, the pristine P1HP polymer is synthesized separately under similar reaction conditions but without the inclusion of iodine. In this case, polymerization is induced using ammonium persulfate ((NH_4_)_2_S_2_O_8_) as an alternative oxidizing agent. The reaction is carried out in a hydrochloric acid (HCl) medium with a concentration of 1.0 M, while the monomer concentration is adjusted to 0.06 M. This method results in the formation of pristine P1HP, providing a reference material for evaluating the structural, optical, and electrical differences between the iodine-modified and unmodified polymer systems.

The successful synthesis of WO_3-X_I_X_/P1HP nanocomposite highlights its potential applicability in advanced electronic and energy storage devices. The combination of WO_3-X_I_X_ and P1HP is anticipated to offer improved conductivity, stability, and electrochemical activity, making it a promising materials for use in supercapacitors and other high-performance electronic applications. Further investigations will focus on optimizing the synthesis conditions and characterizing the material’s electrical, optical, and structural properties to explore its full potential in practical applications.

### Fabrication of pseudosupercapacitors based on WO_3-X_I_X_/P1HP nanocomposite

The fabrication of the pseudosupercapacitor is carried out using the synthesized WO_3-X_I_X_/P1HP nanocomposite as the primary electrode material. To prepare the electrode paste, 0.04 g of the WO_3-X_I_X_/P1HP composite is mixed with 0.005 g of graphite powder, which serves as a conductive additive to enhance electron transfer. This mixture is then dispersed in 0.75 mL of ethanol to ensure uniform suspension. To improve adhesion and conductivity, 0.1 mL of Nafion solution is added, and the resulting dispersion is stirred continuously for 48 h to obtain a highly homogeneous paste.

Once the paste achieves a uniform consistency, it is carefully cast onto a graphite sheet with a total surface area of 1.0 cm^2^. This coated graphite sheet serves as the working electrode in a three-electrode electrochemical cell. The other two electrodes in the system include a saturated calomel electrode (SCE) as the reference electrode and a graphite electrode as the counter electrode. These electrodes are assembled within the electrochemical cell and connected to a CHI electrochemical workstation, which is used for evaluating the electrochemical performance of the pseudosupercapacitor (Fig. 1).

To assess the supercapacitive properties, various electrochemical measurements are conducted, including cyclic voltammetry (CV) and galvanostatic charge–discharge (GCD) analysis. The specific capacitance (Cs) is determined using the current density (I/m), discharge time (Δt), and voltage window (ΔV) as estimated in Eq. 1^3^. Additionally, the energy density (E) is calculated using Eq. 2^3^, where Cs is a key parameter, while the power density (P) is estimated using Eq. 3^3^, which accounts for the energy stored per unit time. These calculations provide critical insights into the energy storage capability and efficiency of the WO_3-X_I_X_/P1HP-based pseudosupercapacitor, making it a promising candidate for advanced energy storage applications.

1 $${C}_{s}=I.\Delta t/ \Delta V.m$$

2 $$E=0.5{\text{C}}_{\text{s}}. ({V}_{max }^{2}-{V}_{min }^{2})$$

3 $$P=E/\Delta t$$

## Results and discussion

### Analyses

**Fig. 2:**
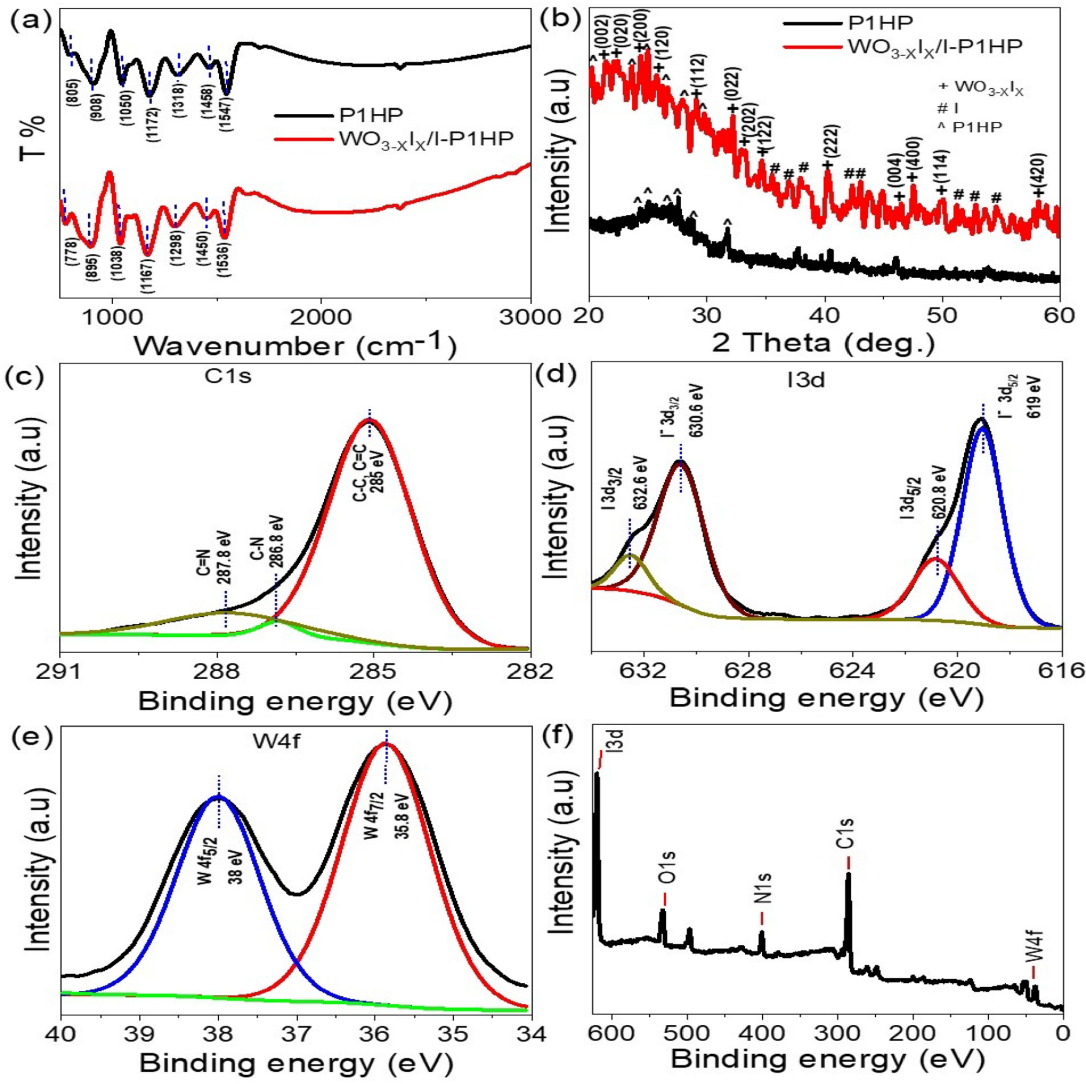
Structural and chemical characterization of the WO_3-X_I_X_/P1HP composite: (**a**) FTIR spectra illustrating vibrational features confirming hybrid formation; (**b**) XRD patterns showing crystalline phases of the composite; XPS analysis of (**c**) C 1 s spectrum, (**d**) I 3d region, (**e**) W 4f. core levels, and (**f**) full elemental survey confirming elemental composition and chemical states.

Figure 2 presents a comprehensive structural and chemical analysis of the WO_3-X_I_X_/P1HP hybrid material, utilizing FTIR, XRD, and XPS. These complementary techniques elucidate the successful synthesis and integration of the inorganic and organic components, confirming the formation of a chemically bonded and structurally coherent hybrid system. The FTIR spectra provide evidence of chemical interactions between the polymer and the inorganic oxide. The pristine P1HP spectrum (black) exhibits distinct vibrational bands at approximately 1547, 1458, and 1318 cm^−1^, which correspond to aromatic C=C stretching, C–C skeletal vibrations, and C–N bond stretching, respectively^18^. Upon the formation of the WO_3-X_I_X_/P1HP hybrid (red spectrum), these bands undergo noticeable shifts and broadening, indicative of strong chemical interactions and perturbations in the local electronic environment due to hybrid formation. Furthermore, the emergence of a peak around 778 cm^−1^ can be attributed to the W–O–W stretching modes associated with the incorporation of the WO_3-X_I_X_ moiety into the polymeric matrix, confirming the establishment of hybrid bonding at the molecular level.

XRD analysis further substantiates the formation of the hybrid composite by revealing a significant enhancement in crystallinity. The P1HP polymer displays a broad halo pattern, indicative of its semi-crystalline nature with weak diffraction peaks observed in the 24–32° range. In contrast, the WO_3-X_I_X_/P1HP composite exhibits sharp and well-defined peaks corresponding to the monoclinic WO_3-X_I_X_ phase. These reflections appear at 24.4°, 32.3°, 34.7°, 40.2°, 47.4°, and 58.2°, indexed to the (200), (022), (122), (222), (400), and (420) crystallographic planes, respectively. Additional peaks at 21.4°, 22.3°, 25.7°, 29.1°, 33.1°, 46.4°, and 50.0° are indexed to (002), (020), (120), (112), (202), (004), and (114), respectively, and match well with the standard JCPDS card no. 83-0950^19^. The presence of additional diffraction peaks is also consistent with iodide phases, suggesting their successful incorporation and enhancing the overall crystallinity and structural integrity of the composite.

XPS spectra offer detailed insights into the electronic structure and chemical states of the composite components. The high-resolution C 1s spectrum shows peaks at 285.1 eV, 286.8, and 287.8 eV are assigned to C–C/C=C, C–N, and C=N bonds, respectively. In the hybrid, these peaks shift, indicating charge transfer or coordination interactions between the polymer and the WO_3-X_I_X_ domains. The I 3d spectrum reveals two doublets at 619.0 eV and 620.8 eV, corresponding to iodide and molecular iodine, respectively, signifying dual charge states of iodine species interacting within the polymer framework. The W 4f. spectrum displays prominent doublets at ~ 35.8 eV (W 4f._7/2_) and ~ 38 eV (W 4f._5/2_), characteristic of W⁶⁺ in sub-stoichiometric tungsten oxide (WO_3-X_), confirming oxygen vacancy-related states. The overall XPS survey scan detects W, O, I, C, and N, validating the material’s composition and purity. Together, these characterizations confirm the successful synthesis of a WO_3-X_I_X_/P1HP composite with enhanced structural ordering and strong chemical interactions, positioning it as a promising material for advanced electrochemical or catalytic applications.

The elemental composition of the composite was determined from the XPS analysis, which revealed weight ratios of W (1.28%), O (12.17%), and I (4.04%). Based on these values, the stoichiometry of the tungsten oxide iodide phase is estimated to correspond to WO_2.6_I_0.4_, indicating partial substitution of oxygen by iodine. This iodinated tungsten oxide framework is homogeneously integrated within the P1HP matrix, forming the WO_2.6_I_0.4_/P1HP nanocomposite with strong interfacial coupling between the inorganic and polymeric components.

**Fig. 3:**
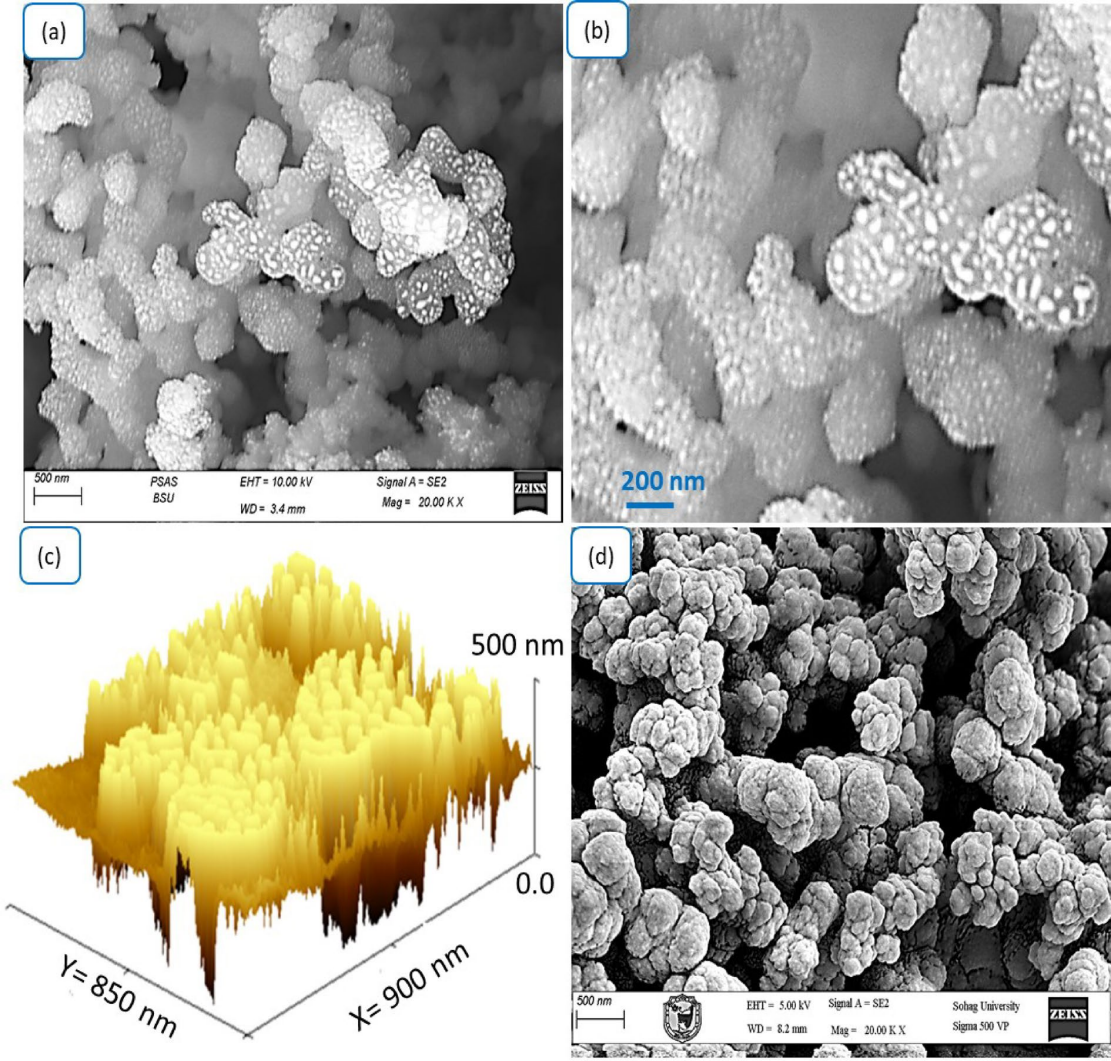
Surface morphology and topographical features of the WO_3-X_I_X_/P1HP nanocomposite: (**a**, **b**) SEM images captured at different magnifications and (**c**) 3D surface profile showing cross-sectional topography. while (**d**) SEM of the pristine P1HP.

Figure 3 presents a comprehensive morphological evaluation of the WO_3-X_I_X_/P1HP composite using SEM at different magnifications and a 3D surface profile analysis. The observations provide critical insights into the surface texture, particle distribution, and structural evolution upon hybrid formation. Figure 3a and b show high-resolution SEM images of the WO_3-X_I_X_/P1HP composite at increasing magnifications. The morphology reveals densely packed, cauliflower-like nanostructures with well-developed surface roughness and hierarchical texture. These structures are composed of smaller aggregated nanoparticles, forming interconnected networks that exhibit a high surface-to-volume ratio^20–22^. The bright contrast regions observed in these images are likely associated with the inclusion of the tungsten oxide and iodide components, which are embedded uniformly within the polymer matrix with an average particle size of 50nm. The hybridization appears to enhance the structural integrity and uniformity of the material, leading to the formation of highly textured and porous domains. Such morphological features are advantageous for applications requiring enhanced surface reactivity, such as catalysis, sensing, or electrochemical energy storage.

Figure 3c provides a 3D surface topography of the WO_3-X_I_X_/P1HP composite, displaying vertical and lateral dimensions at the nanometer scale. The cross-sectional analysis confirms the significant surface roughness, with height variations reaching approximately 500 nm over an area of 900 × 850 nm^2^, in which the estimated individual particle has an average size of 30 nm. The presence of vertically oriented nanostructures and sharp protrusions further supports the SEM findings, reinforcing the high surface area and nanoscale texturing of the composite. This hierarchical architecture is indicative of a favorable environment for charge transport, ion diffusion, and increased active site accessibility, essential for functional nanomaterial systems.

Figure 3d illustrates the SEM image of the pristine P1HP polymer. Compared to the hybrid composite, the P1HP exhibits a more compact and relatively smoother morphology, characterized by spherical and partially agglomerated grains. The surface is less structured, with minimal porosity and lower surface complexity. This stark contrast highlights the morphological transformation induced by the incorporation of WO_3-X_ and iodide phases into the polymer framework. The transformation from isolated polymer grains to a well-interconnected, rough, and porous architecture confirms the successful formation of the hybrid material and its enhanced structural features. So, the morphological analysis confirms that the incorporation of WO_3-X_I_X_ within the P1HP matrix significantly alters the surface characteristics, promoting the development of a textured, porous, and nanostructured hybrid. This evolution in morphology is crucial for the material’s potential utility in various advanced applications, including photocatalysis, supercapacitors, and hybrid electrochemical systems.

The TEM image distinctly illustrates the intimate interfacial coupling between WO_3-X_I_X_ and the P1HP matrix, which is estimated in Figure S1a and b at various magnifications. The variation in contrast across the micrograph signifies the homogeneous dispersion of WO_3-X_I_X_ nanocrystals within the polymer framework. This nanoscale integration facilitates efficient structural coherence between the inorganic and organic phases. The observed crystalline domains, averaging around 30 nm, further confirm the formation of a finely organized hybrid architecture.

### Electrochemical characterization of the WO_3-X_I_X_/P1HP nanocomposite-based pseudosupercapacitor

The electrochemical performance of the WO_3-X_I_X_/P1HP nanocomposite-based pseudosupercapacitor is systematically investigated using a conventional three-electrode configuration. In this setup, a composite paste coated onto a graphite sheet (with an active surface area of 1.0 cm^2^) served as the working electrode. The selection of 1.0 M HCl as the electrolyte was based on its superior ionic conductivity and ability to provide a high concentration of protons, which effectively facilitates the redox transitions between the oxidation states of tungsten (W^6+^/W^5+^/W^4+^) and the polymer backbone. The acidic medium promotes rapid ion diffusion and charge compensation within the WO_3-X_I_X_/P1HP nanocomposite, thereby enhancing its specific capacitance and rate performance.

A saturated calomel electrode was utilized as the reference electrode, ensuring stable and reliable potential control throughout the measurements. Electrochemical assessments were conducted within a potential window ranging from 0.0 to 1.0 V to evaluate the charge–discharge behavior of the device.

Furthermore, cyclic voltammetry analyses were performed at various scan rates to elucidate the redox characteristics and capacitive behavior of the pseudosupercapacitor. The integrated area under the CV curves was used to quantify the contribution of faradaic processes and to estimate the charge storage capacity of the nanocomposite electrode under dynamic conditions.

**Fig. 4:**
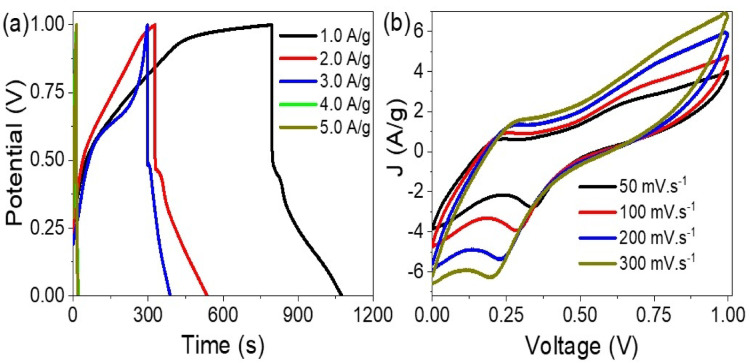
Electrochemical performance of the WO_3-X_I_X_/P1HP nanocomposite-based pseudosupercapacitor: (**a**) galvanostatic charge–discharge profiles at different current densities and (**b**) cyclic voltammetry curves recorded at varying scan rates.

The electrochemical behavior of the pseudosupercapacitor constructed from the WO_3-X_I_X_/P1HP nanocomposite was evaluated via galvanostatic charge–discharge (GCD) and cyclic voltammetry (CV) techniques, as illustrated in Fig. 4. These analyses provide comprehensive insights into the charge storage capabilities and rate-dependent kinetics of the electrode material. Figure 4a presents the GCD curves recorded at varying current densities ranging from 1.0 to 5.0 A/g. The profiles exhibit quasi-linear and symmetric charge–discharge characteristics, indicative of a predominant pseudocapacitive behavior governed by faradaic redox reactions. Notably, as the applied current density increases, the discharge time significantly decreases. At 1.0 A/g, the discharge duration is the longest, reflecting a high charge storage capacity. Conversely, at higher current densities (e.g., 5.0 A/g), the discharge time is considerably shortened. This trend can be attributed to the limited diffusion of protons into the electrode matrix at higher current rates, where the electrochemical reactions occur more rapidly, and ion transport becomes a limiting factor^23,24^. Consequently, a reduced fraction of the active sites participates in the redox reactions, leading to diminished charge storage at elevated current densities^25–27^.

Figure 4b depicts the CV curves at scan rates ranging from 50 to 300 mV/s. The voltammograms retain their characteristic pseudocapacitive shape, featuring broad, symmetrical redox peaks that arise from the reversible faradaic transitions of tungsten oxide species. As the scan rate increases, the peak current also rises, consistent with the electrochemical kinetics of pseudocapacitive materials. At slower scan rates (e.g., 50 mV/s), the ions have sufficient time to access the full surface area of the active material, thereby enabling deeper intercalation and more efficient redox processes.

The distinct oxidation and reduction peaks observed in the CV profiles correspond to the reversible redox transitions of tungsten ions within the WO_3-X_I_X_ framework, coupled with proton-coupled electron transfer processes facilitated by the conductive P1HP polymer matrix. Specifically, the anodic peaks can be attributed to the oxidation of W^5^⁺ → W^6^⁺, while the cathodic peaks arise from the reverse reduction process (W^6^⁺ → W^5^⁺/W^4^⁺). The iodide species further contribute by mediating charge compensation and enhancing electron mobility during these redox transitions. Simultaneously, the doping/de-doping of protons and chloride/iodide anions within the polymer backbone (P1HP) reinforces pseudocapacitive behavior through additional reversible redox reactions on the nitrogen and sulfur sites of the polymer. The combined effect of these tungsten-centered and polymer-assisted redox processes results in the broad, well-defined redox peaks observed in the CV curves, confirming the synergistic charge storage mechanism operative in the WO_3-X_I_X_/P1HP nanocomposite electrode. So, the WO_3-X_I_X_/P1HP nanocomposite demonstrates excellent pseudocapacitive behavior characterized by reversible redox activity and efficient charge storage. However, the performance is influenced by both current density and scan rate, with optimal behavior observed at lower rates where ion diffusion and faradaic interactions are maximized^28,29^.

**Fig. 5:**
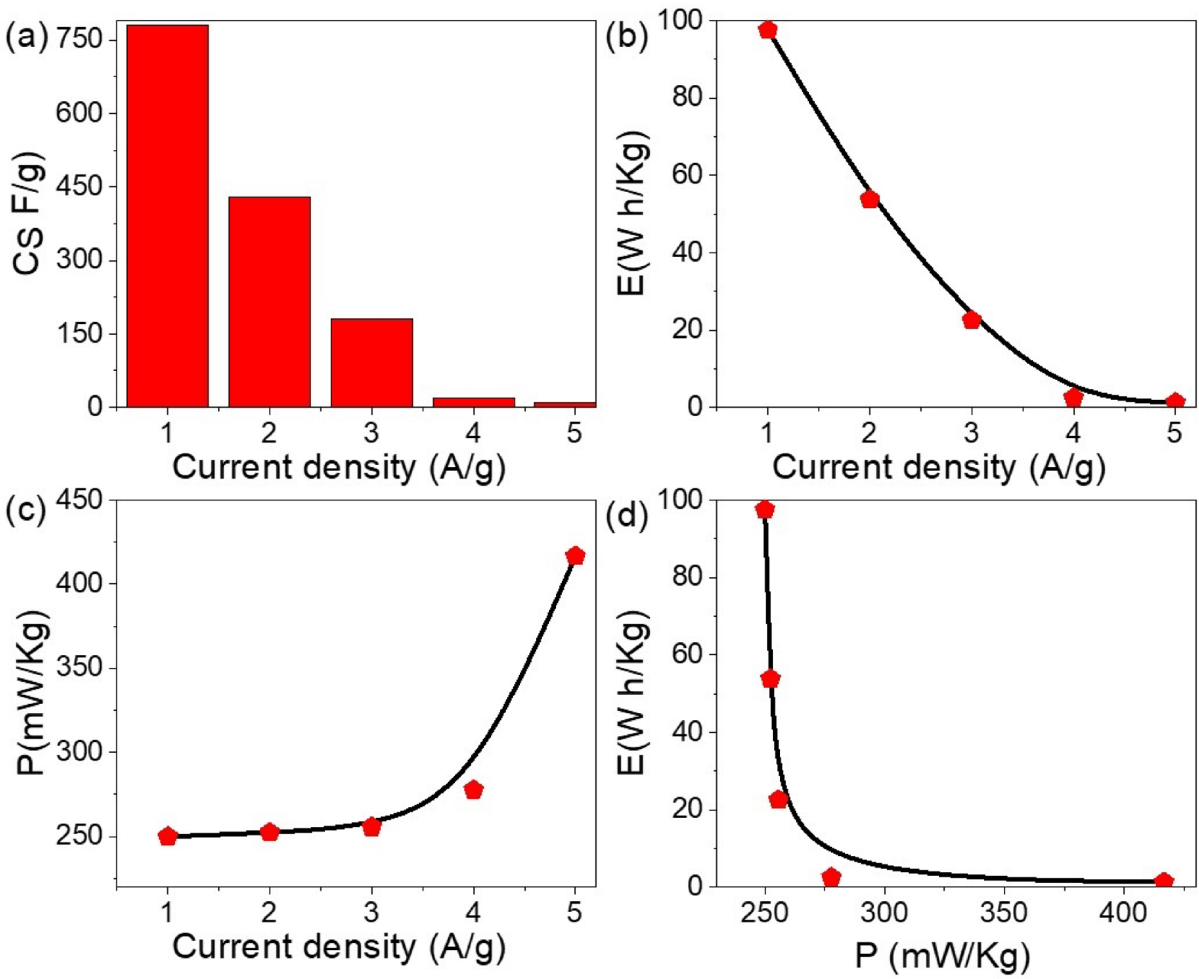
Electrochemical performance of the WO_3-X_I_X_/P1HP nanocomposite-based pseudosupercapacitor: (**a**) CS with current densities, (**b**) Energy density, (**c**) Power density, and (**d**) Ragone plot based on Galvanostatic charge–discharge.

The electrochemical performance of the WO_3-X_I_X_/P1HP nanocomposite-based pseudosupercapacitor was systematically investigated to assess its charge storage capabilities. The specific capacitance (Cs) was determined using Eq. 1 and the galvanostatic charge–discharge (GCD) profiles illustrated in Fig. 5a. The Cs values were calculated based on the discharge time (Δt), applied voltage window (ΔV), and the mass of the active nanocomposite material. The variation of Cs as a function of current density revealed an optimal value of 775 F/g at a current density of 1.0 A/g. As the current density increased to 2.0 A/g, the specific capacitance decreased to 425 F/g, which remains relatively high, highlighting the superior charge storage capability of the WO_3-X_I_X_/P1HP hybrid system.

These excellent capacitance values underscore the efficient pseudocapacitive behavior of the device, which is likely attributed to the facile insertion and de-insertion of ions into the porous nanostructure of the active material. The synergy between the metal oxide framework and the polymeric matrix enhances ionic mobility and electron transport, thereby improving the overall electrochemical response.

The corresponding energy density (E) values were further evaluated using Eq. 2 and are presented in Fig. 5b. As the current density was varied from 1.0 to 5.0 A/g, the energy density decreased in accordance with the expected trend, with optimal values reaching 100 Wh/kg at lower current densities and reducing to 52 Wh/kg at higher rates. These values indicate that the device retains considerable energy even under high power operating conditions.

Furthermore, the power density (P) was calculated using Eq. 3 and is shown in Fig. 5c. The maximum power density attained was 420 mW/kg at a high current density of 5.0 A/g, while a notable value of 250 mW/kg was recorded at 1.0 A/g. These results demonstrate the device’s ability to deliver energy rapidly, which is essential for high-performance energy storage systems.

To comprehensively evaluate the energy and power performance of the pseudosupercapacitor, a Ragone plot was constructed (Fig. 5d). The plot illustrates the relationship between energy and power density, confirming the remarkable performance of the WO_3-X_I_X_/P1HP supercapacitor, with a maximum energy density of 100 Wh/kg and a power density of 250 mW/kg. These findings confirm the potential of this nanocomposite system for advanced energy storage applications.

**Fig. 6:**
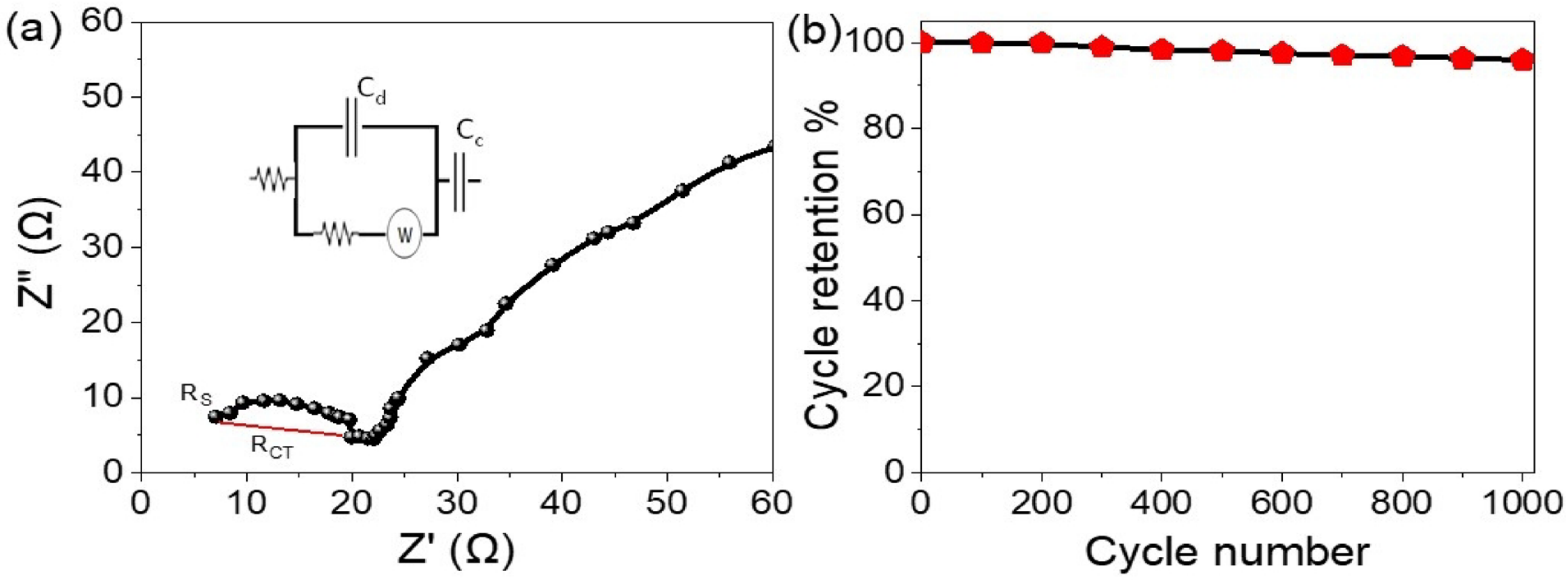
(**a**) Electrochemical impedance spectroscopy analysis and (**b**) long-term cycling stability of the pseudosupercapacitor system constructed from the WO_3-X_I_X_/P1HP nanocomposite.

The electrochemical resistance characteristics of the WO_3-X_I_X_/P1HP nanocomposite-based pseudosupercapacitor were assessed using electrochemical impedance spectroscopy (EIS)^1,30^, as illustrated in Fig. 6a. The equivalent series resistance (Rs)^31–33^, representing the total internal resistance of the device—including contributions from the electrolyte, electrode material, and contact interfaces—is found to be approximately 6.5 Ω. This relatively low Rs value indicates excellent electronic conductivity and efficient energy transfer, which is crucial for achieving high power density.

Additionally, the charge transfer resistance (R_CT_), which is indicative of the redox kinetics at the electrode–electrolyte interface, was estimated to be 13.5 Ω. This value, derived from the diameter of the semicircle in the Nyquist plot^34,35^, reflects the favorable faradaic behavior of the device. The low Rct suggests fast ion transport and efficient charge storage^31^, confirming the outstanding pseudocapacitive performance of the WO_3-X_I_X_/P1HP nanocomposite.

To further evaluate the operational stability, cyclic charge–discharge tests were conducted, with the results presented in Fig. 6b. Remarkably, the device retained 95.5% of its initial capacitance after 1000 cycles, demonstrating excellent long-term electrochemical stability. This high retention rate indicates minimal degradation of the electrode material and highlights its robust cycling behavior under continuous operation.

Figure S2 presents the calculated coulombic efficiency of the WO_3-X_I_X_/P1HP-based pseudosupercapacitor at various current densities ranging from 1 to 5 A g^−1^. The device exhibits excellent electrochemical reversibility, as reflected by the gradual increase in coulombic efficiency with increasing current density. This trend indicates that the charge–discharge processes become more stable and kinetically favorable at higher current loads, likely due to enhanced ion diffusion and reduced side reactions within the polymer/oxide interface^36,37^. Notably, the coulombic efficiency reaches approximately 91.5% and 92% at 4 and 5 A g^−1^, respectively, demonstrating the superior structural stability and fast charge-transfer capability of the WO_3_–Iₓ/P1HP nanocomposite electrode during prolonged cycling.

A comparative assessment presented in Table 1 clearly highlights the superior electrochemical behavior of the WO_3-X_I_X_/P1HP pseudosupercapacitor relative to recently reported WO_3_ and polymer-based systems. The significantly higher specific capacitance, enhanced rate capability, and outstanding long-term cycling stability demonstrate the efficiency of the tungsten oxide iodide–polymer interface in facilitating rapid charge transfer and reversible redox processes. These collective advantages affirm that the designed WO_3-X_I_X_/P1HP nanocomposite outperforms many previously developed pseudosupercapacitors, establishing it as a highly competitive and advanced material for next-generation energy storage applications.

**Table 1:** Comparative analysis of the capacitance performance of the WO_3-X_I_X_/P1HP-based pseudosupercapacitor system in relation to previously reported studies.

Supercapacitor electrode	C_S_ (F/g)	Electrolyte	Current density A/g
NiMoO_4_ /WO_3_ nanoflowers on Ni foam^38^	429	KOH	1
WO_3_/PVA-H_2_SO_4_/PANI device^39^	484	PVA-H_2_SO_4_ gel	1
WO_3_-carbon^40^	214	H_2_SO_4_	0.5
WO_3-X_^41^	380	H_2_SO_4_	1
WO_3_-RGO^42^	495	H_2_SO_4_	0.5
WO_3_-Graphene^43^	143	H_2_SO_4_	0.1
MnO_2_-Mn_2_O_3_/Poly-2-methylaniline^15^	72	1.0 M HCl	0.2
polyaniline/silver oxide/silver^16^	4.0	1.0 M NaOH	0.2
CoO-CuO/G-C3N4^17^	65	6.0 M KOH	0.5
Fe_2_O_3_/poly-2-aminothiophenol^9^	44.5	1.0 M NaOH	0.2
β-Ni(OH)_2_^44^	14.2	1.0 M NaOH	1.0
Polypyrrole-Ni(OH)_2_^45^	70	poly(vinyl alcohol)/H_3_PO_4_	0.005
NiO/Ni(OH)_2_^46^	–	1.0 M KOH	–
ZnS–ZnO/g-C_3_N_4_^47^	15	1.0 M Na_2_SO_4_	0.2
β-Ni(OH)_2_ /G-C_3_N_4_^44^	20.5	1.0 M NaOH	1.0
Gd/G-C_3_N_4_^48^	16	poly(vinyl alcohol)/H_3_PO_4_	–
WO_3-X_I_X_/P1HP nanocomposite (this work)	775	1.0 M HCl	1.0

## Conclusions

A novel WO_3-X_I_X_/P1HP nanocomposite pseudosupercapacitor has been successfully developed and comprehensively evaluated using a three-electrode configuration. Structural and spectroscopic analyses (XRD and XPS) confirmed the uniform incorporation of iodide within the tungsten oxide lattice and along the poly(1H-pyrrole) framework, forming a sub-stoichiometric WO_3-X_ phase rich in redox-active centers. This tailored architecture enhances electronic coupling and accelerates proton and electron transport across the hybrid interface. The synergistic interplay between the inorganic WO_3-X_I_X_ and the conductive P1HP polymer, together with the composite’s hierarchical morphology, underpins its exceptional electrochemical response. The electrode achieved a specific capacitance of 775 F g^−1^ at 1.0 A g^−1^ and maintained 425 F g^−1^ at 2.0 A g^−1^, corresponding to energy densities of 100 Wh kg^-1^ and 52 Wh kg^−1^, respectively. These values surpass many recently reported WO_3_^−^ and polymer-based systems, demonstrating the efficiency of the iodide-mediated redox mechanism and the optimized charge transport pathways within the nanocomposite. Although this study primarily employs a three-electrode configuration to elucidate the intrinsic electrochemical behavior, the results lay a strong foundation for future device-level evaluations. Ongoing work will focus on scaling the architecture into two-electrode and flexible configurations to assess practical feasibility. Overall, the WO_3-X_I_X_/P1HP hybrid represents a promising next-generation electrode material, bridging the gap between laboratory-scale performance and future integration into advanced, sustainable energy storage technologies.

## Supplementary Information

Below is the link to the electronic supplementary material.

Supplementary Material 1

## Data Availability

The datasets used and/or analyzed during the present study are available from the corresponding author upon reasonable request. All data generated or analyzed during this study are included in this published article.
