# Supplementary material for: High-performance supercapacitor based on tungsten oxide iodide/polymer nanocomposite for advanced energy storage
Source: Sci Rep. 2025 Dec 4;16:410. doi: 10.1038/s41598-025-29780-y (PMC12769496; doi:10.1038/s41598-025-29780-y)
Supplement: Supplementary file 1 — Supplementary Material 1 [file 41598_2025_29780_MOESM1_ESM.docx]

**High-Performance Supercapacitor Based on Tungsten Oxide Iodide/Polymer Nanocomposite for Advanced Energy Storage**

**Ahmed H. AbdEl-Salam^1*^, Hassan A. Ewais^2,3^, Mohamed Rabia^3^, Min Liu^4^ and Yasser M. Al Angari^2^**

*^1^* *Chemistry Department, Faculty of Science, University of Jeddah, Jeddah, Saudi Arabia*

*^2^ Chemistry Department, Faculty of Science, King Abdulaziz University, P.O. Box.80200, Jeddah 21589, Saudi Arabia*

*^3^ Nanomaterials Science Research Laboratory, Chemistry Department, Faculty of Science, Beni-Suef University, Beni-Suef, 62514, Egypt*

*^4^ School of Physics, Central South University, Changsha 410075, China*

*Corresponding author: Ahmed H. AbdEl-Salam; [anooreldeen@uj.edu.sa](mailto:anooreldeen@uj.edu.sa)


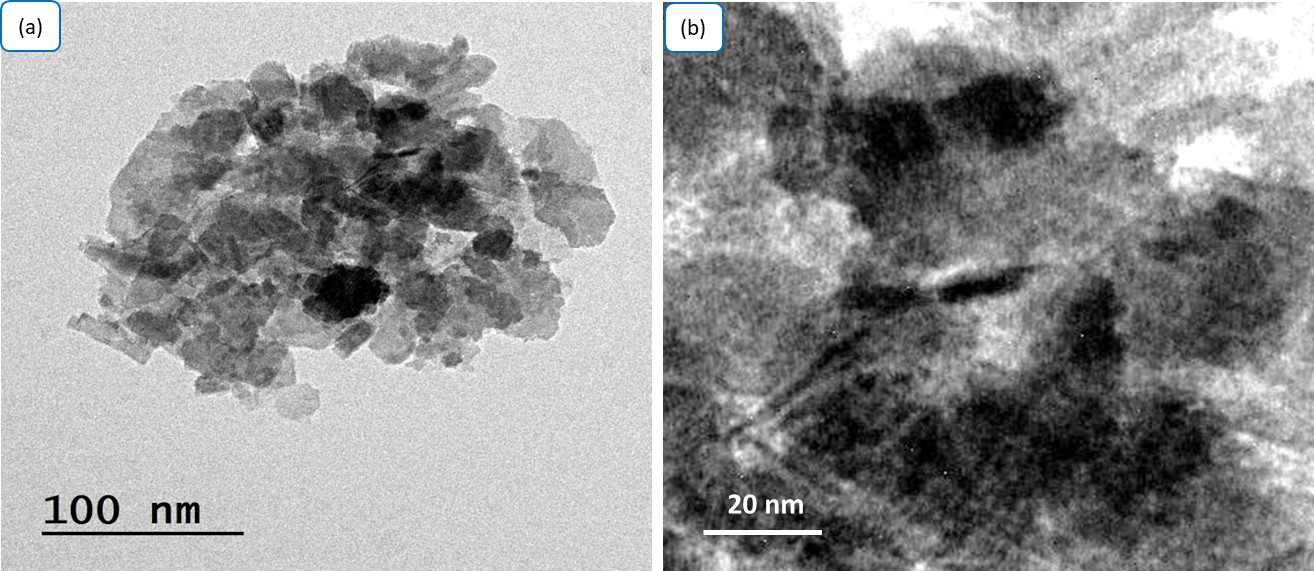


**Figure S1.** (a,b) TEM micrographs of the WO_3-X_I_X_/P1HP nanocomposite captured at different magnifications, revealing the uniform dispersion and nanoscale morphology of the hybrid structure.


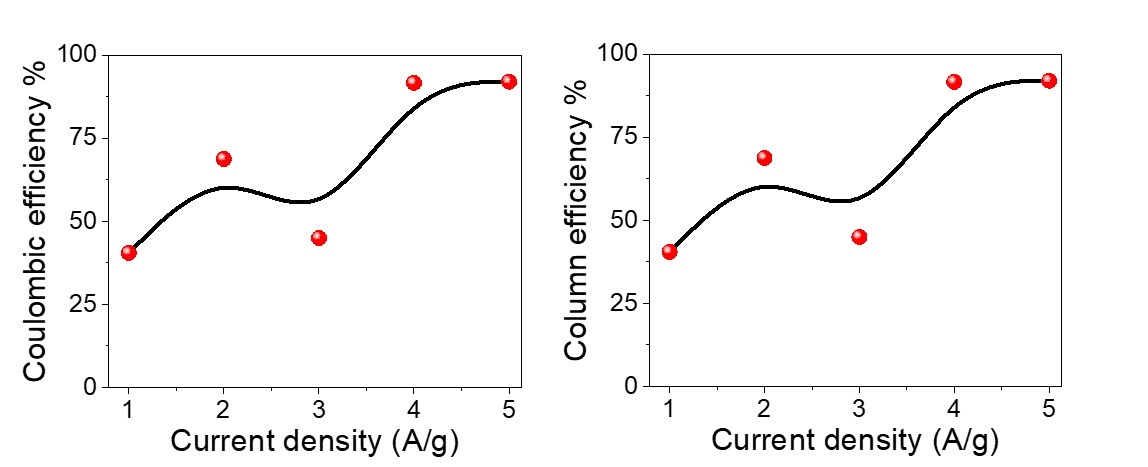


Figure S2. The estimated coulombic efficiency for the fabricated pseudosupercapacitor based on WO_3-X_I_X_/P1HP nanocomposite at various current densities.
